# Biogenic Nanoparticles Silver and Copper and Their Composites Derived from Marine Alga *Ulva lactuca*: Insight into the Characterizations, Antibacterial Activity, and Anti-Biofilm Formation

**DOI:** 10.3390/molecules28176324

**Published:** 2023-08-29

**Authors:** Ragaa A. Hamouda, Mada A. Alharthi, Amenah S. Alotaibi, Asma Massad Alenzi, Doha A. Albalawi, Rabab R. Makharita

**Affiliations:** 1 Department of Biology, College of Sciences and Arts Khulais, University of Jeddah, Jeddah 21959, Saudi Arabia; 2 Department of Microbial Biotechnology, Genetic Engineering and Biotechnology Research Institute, University of Sadat City, Sadat City 32897, Egypt; 3 Genomic & Biotechnology Unit, Department of Biology, Faculty of Science, University of Tabuk, Tabuk 71491, Saudi Arabia; 4 Botany and Microbiology Department, Faculty of Science, Suez Canal University, Ismailia 41522, Egypt

**Keywords:** *Ulva lactuca*, Ag nanoparticles, copper nanoparticles, nanocomposites, antibacterial

## Abstract

Bacterial pathogens cause pain and death, add significantly to the expense of healthcare globally, and pose a serious concern in many aspects of daily life. Additionally, they raise significant issues in other industries, including pharmaceuticals, clothing, and food packaging. Due to their unique properties, a great deal of attention has been given to biogenic metal nanoparticles, nanocomposites, and their applications against pathogenic bacteria. This study is focused on biogenic silver and copper nanoparticles and their composites (UL/Ag_2_ O-NPS, Ul/CuO-NPs, and Ul/Ag/Cu-NCMs) produced by the marine green alga *Ulva lactuca*. The characterization of biogenic nanoparticles UL/Ag_2_ O-NPS and Ul/CuO-NPs and their composites Ul/Ag/Cu-NCMs has been accomplished by FT-IR, SEM, TEM, EDS, XRD, and the zeta potential. Minimum inhibitory concentration (MIC) and minimum bactericidal concentration (MBC) experiments were conducted to prove antibacterial activity against both Gram-positive and Gram-negative bacteria and anti-biofilm. The FTIR spectroscopy results indicate the exiting band at 1633 cm^−1^, which represents N–H stretching in nanocomposites, with a small shift in both copper and silver nanoparticles, which is responsible for the bio-reduction of nanoparticles. The TEM image reveals that the Ul/Ag/Cu-NCMs were hexagonal, and the size distribution ranged from 10 to 35 nm. Meanwhile, Ul/CuO-NPs are rod-shaped, whereas UL/Ag_2_ O-NPS are spherical. The EDX analysis shows that Cu metal was present in a high weight percentage over Ag in the case of bio-Ag/Cu-NCMs. The X-ray diffraction denotes that Ul/Ag/Cu-NCMs, UL/CuO-NPs, and UL/Ag_2_ O-NPS were crystalline. The results predicted by the zeta potential demonstrate that Ul/Ag/Cu-NCMs were more stable than Ul/CuO-NPs. The antibacterial activity of UL/Ag_2_ O-NPS, Ul/Ag/Cu-NCMs, and UL/CuO-NPs was studied against eleven Gram-negative and Gram-positive multidrug-resistant bacterial species. The maximum inhibition zones were obtained with UL/Ag_2_ O-NPS, followed by Ul/Ag/Cu-NCMs and Ul/CuO-NPs in all the tested bacteria. The maximum anti-biofilm percentage formed by *E. coli* KY856933 was obtained with UL/Ag_2_ O-NPS. These findings suggest that the synthesized nanoparticles might be a great alternative for use as an antibacterial agent against different multidrug-resistant bacterial strains.

## 1. Introduction

Nanotechnology, in combination with biology, provides a promising field of nanobiotechnology. This includes living entities, such as cyanobacteria, bacteria, viruses, algae, actinomycetes, yeasts, fungi, and plants [1,2,3]. The green biogenesis of nanoparticles (NPs) utilizing either algae or plant extracts has appeared to be an economical and environmentally beneficial technique [4,5,6]. Specifically, macro-algae cover various bioactive contents with multiple constructions and promising biological applications [7,8]. Due to their potential utility in the bio-manufacture of nanomaterials, algae have been referred to as “nanofactories”. The main benefit of this environmentally friendly fabrication is the lack of hazardous solvents and reagents, which prevents the production of waste and byproducts during the synthesis of metal nanoparticles. In addition, the use of algae fosters the goal of using natural, renewable resources [9,10]. The process of metal stabilization and reduction is supported by a variety of substances found in the crude extracts of macroalgae, such as amines, amides, alkaloids, terpenoids, pigments, proteins, etc. [11,12]. *Ulva lactuca* is a type of green seaweed comprised of highly active substances with antitumor, antioxidant, hypocholesterolemic, and antimicrobial capabilities [13]. Ag-NPs can be fabricated using *U. lactuca* [14]. Ag-NPs fabricated using *U. lactuca* have antibacterial activities against *Escherichia coli* and *Pseudomonas aeruginosa* [15]. The biosynthesis of nanoparticles by *U. lactuca* is a reliable, economical, and eco-friendly process for the synthesis of metallic nanoparticles [16].

A nanocomposite is a composite material in which at least one of the materials is one dimension in size, around 10–9 nm [17]. They have high-performance materials that exhibit rare characteristics. Their perspective is so notable that they are valuable in many fields [18], from packaging to biomedical applications [19]. The antifungal effect resulting from the copper–silver–chitosan nanocomposite was more significant than that of other nanoparticles tested, which has had an essential influence on the growth of *Candida albicans* in laboratory conditions compared to other nanoparticles [20]. Different studies showed that the as-synthesized Ag/Cu-NMCs could be an efficient substitute for an antimicrobial agent against drug-resistant microbes and plant-pathogenic bacteria [21,22]. The Ag–Cu–Co oxide caused a maximum zone of inhibition against *E. coli* that was found to be 25 mm [23]. The fabricated nanostructured surfaces (Ag–CuxO nanostructures) exhibit excellent bactericidal efficiency against *E. coli* (Gram-negative) and *Staphylococcus aureus* (Gram-positive) [24]. Good antibacterial activity was noted against both bacteria in milk (*Escherichia coli* and *Staphylococcus aureus*) with a sensitivity of 40–90% at CuO/Ag concentrations ranging from 6 to 200 mg/100 mL [25]. CuO/Ag_2_O NCPs synthesized by the leaf extract of *Eichhornia crassipes* show good antibacterial activities against four human pathogenic bacteria, such as *K. pneumonia*, *E. coli*, *S. epidermides*, and *S. aureus* [26].

Due to their unique characteristics, such as superior electrical conductivity, low electrochemical migration behavior, a high melting point, and, most importantly, low cost, copper nanoparticles have applications in many areas, such as industries, medicine, electronics, etc. [27,28]. For more than 200 years, copper has been known for its ability to inhibit the growth of microorganisms and lower microbial concentrations by 99.9% [29,30]. Numerous studies have shown that copper nanoparticles show excellent antimicrobial activity against many bacteria [28,31]. Moreover, nano-copper oxide (Cu-O) works as an essential antimicrobial agent against *Staphylococcus aureus*, *Escherichia coli*, *Vibrio cholera*, *Pseudomonas aeruginosa*, *Bacillus subtilis*, and *Syphillis typhus* [32,33,34,35]. The reputation of copper has expanded lately with the COVID-19 pandemic, when it was noticed that SARS-CoV-2 on copper surfaces decomposes more rapidly (in four hours) than other materials, such as plastic and stainless steel [36].

This study aims to investigate the efficacy of the marine green alga *Ulva lactuca*, collected from the Jeddah seashore, as reducing and stabilizing agents for the biofabrication of UL/Ag_2_ O-NPS, Ul/CuO-NPs, and Ul/Ag/Cu-NMCs. We have compared the characteristics of silver and copper nanoparticles and their composites. We have also determined the antibacterial activities of biofabricated nanoparticles and their composites against some multi-drug-resistance bacterial strains.

## 2. Results and Discussion

### 2.1. Characterisation of Ul/Ag_2_O-NPS, Ul/Ag/Cu-NCMs, and Ul/CuO-NPs

#### 2.1.1. Fourier Transform Infrared (FTIR) of UL/Ag_2_ O-NPS, Ul/Ag/Cu-NCMs and Ul/CuO-NPs

**Figure 1:**
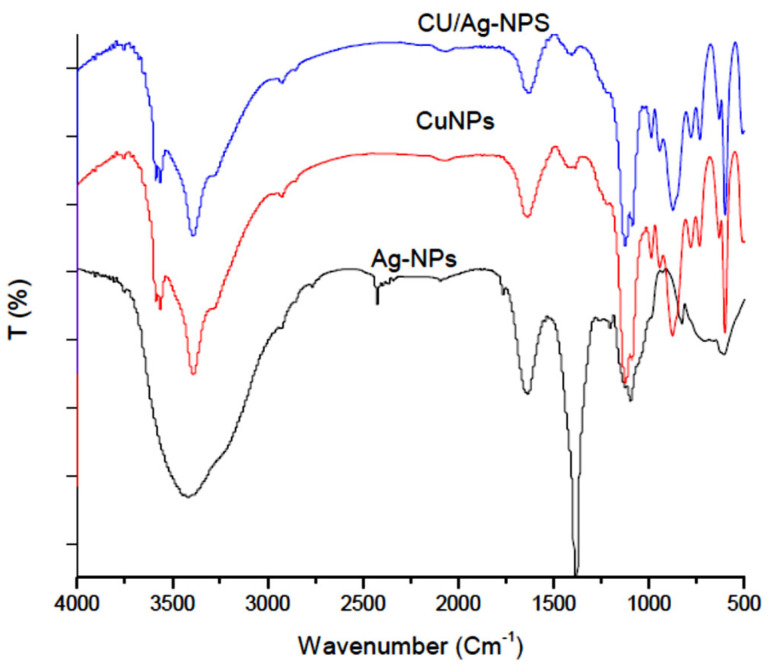
Fourier transform infrared spectroscopic data of Ul/Ag_2_O-NPS, Ul/Ag/Cu-NCMs, and Ul/CuO-NPs derived from *U. lactuca*.

Fourier transform infrared spectroscopy was conducted to reveal the functional groups in the molecules characterized during the production of the newly synthesized nanoparticles, which are responsible for the stabilization and coating of the nanoparticles in all three nanoparticles. The FTIR spectra bands of the silver nanoparticles are shown in Figure 1 and Table 1. The silver nanoparticles (UL/Ag_2_ O-NPS) biofabricated by *U. lactuca* illustrate 10 peaks at 3752, 3419, 2426, 1765, 1636, 1383, 1202, 1097, 826, and 606 cm^−1^. The FT-IR spectroscopy of the Ul/CuO-NPs shows 17 peaks at 3587, 3564, 3390, 2927, 1635, 1386, 1123, 1090, 988, 944, 876, 781, 724, 623, 601, 483, and 420 cm^−1^. The silver/copper nanocomposites exhibit 18 peaks at 3588, 3565, 3390, 2925, 1633, 1125, 1088, 1025, 987, 944, 874, 780, 733, 631, 600, 509, 483 and 417 cm^−1^. The band at 1633 cm^−1^, which is present in all of the synthesized nanoparticles (UL/Ag_2_ O-NPS, Ul/Ag/Cu-NCMs, and Ul/CuO-NPs) with a small shift, represents the N–H stretching band, which denotes the active group that acts as reducing and stabilizing agents in biofabricated nanoparticles. Phytochemical compounds found in biomass, such as alcohols, aldehydes, alkanes, and epoxy groups or ether groups, can be responsible for the nucleation process to reduce Ag+ to Ag° [37]. The biosynthesis of nanoparticles may be triggered by several compounds, such as carbonyl groups, phenolics, flavonones, terpenoids, amides, amines, proteins, pigments, alkaloids, and other reducing agents present in the biological extracts [11].

#### 2.1.2. Scanning and Transmission Electron Microscope (SEM and TEM) of UL/Ag_2_ O-NPS, Ul/CuO-NPs, and Ul/Ag/Cu-NCMs Derived from *U. lactuca*

**Figure 2:**
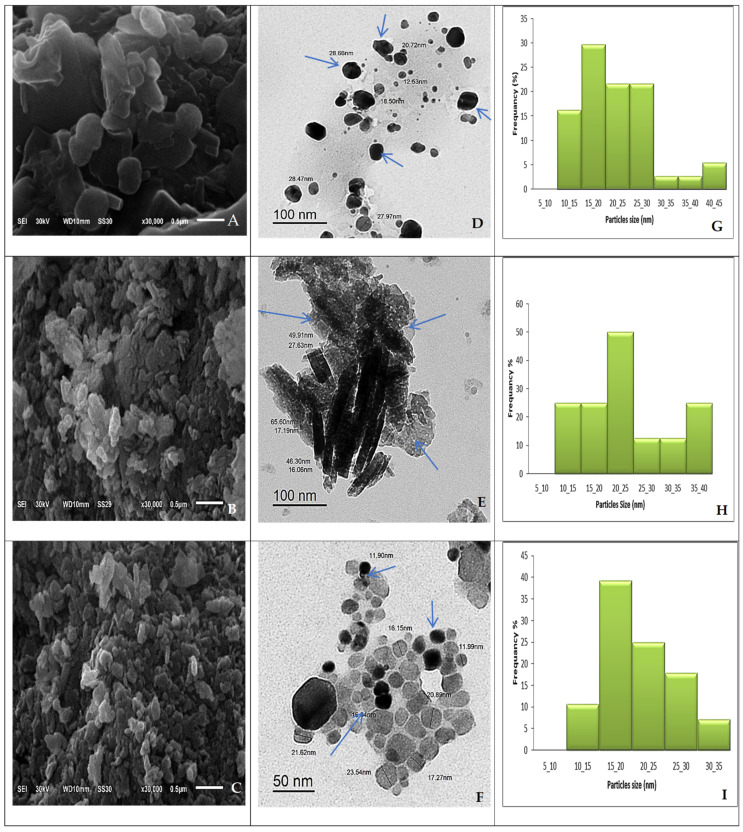
The SEM (**A**–**C**) and TEM (**D**–**F**) images and frequency distribution (%) (**G**–**I**) of UL/Ag_2_ O-NPS, Ul/CuO-NPs, Ul/Ag/Cu-NCMs derived from *U. lactuca.* Arrows refer to the shell around nanoparticles.

The SEM image has been used to illustrate the size and shape of the produced nanoparticles. As shown in the sample images in Figure 2A–C, the surface is rough due to the presence of Cu, Ag, and Ag combined with Cu. The modification in morphology and coarseness of the surface was due to the foundation of nanoparticles [57].

The TEM image shows that UL/Ag_2_O-NPS are spherical in shape and range from 10 to 45 nm (Figure 2D and Table 2). The image proves that UL/Ag_2_ O-NPS are polydispersed and range in size from 15 to 20 nm, as shown in the frequency histogram (Figure 2G). The Ag-NPs synthesized by *Ulva fasciata* were spherical and 50 nm in size [58]; by *Enteromorpha flexuosa* were 2–32 nm/circular [59]; by *Padina tetrastromatica* were 14 nm/spherical [60]; and by *Gracilaria corticata* were 8–46 nm/spherical [61].

The TEM image of the Ul/CuO-NPs shows they are rod-shaped, one-dimensional nanoparticles; the length of the rod ranged from 46 to 65 nm, and the width ranged from 10 to 40 nm (Figure 2E). The Ul/CuO-NPs are polydispersed and range in size, according to width, from 20 to 25 nm (Figure 2E and Table 2). The size of the synthesized copper nanoparticles using *Zingiber officinale* and *Curcuma longa* rhizome extract was in the nano-range of approximately 20 to 100 nm [62], and the average size of the copper nanoparticles synthesized by *Eucalyptus* leaf extract ranged from 10 to 130 nm. In contrast, the size of the mint leaf extracts of the copper nanoparticles ranged from 23 to 39 nm [63]. The results estimated that there was some aggregation of Ul/CuO-NPs; some nanoparticles were well distributed, while most were found in the agglomerated form [63]. The results agree with Ramzan et al. [64] when using *Cedrus deodara* aqueous extract to synthesize the copper nanoparticles; only a few spherical-shaped and well-separated particles were found, and most of them were agglomerated. Amaliyah et al. [65] used *Piper retrofractum* Vahl extract to synthesize spherical Cu-NPs with the propensity to form a random aggregate. Moreover, Mahmoud et al. [66] utilized orange peels to synthesize CuO-NPs, which appear to aggregate. Meanwhile, when Ag and Cu were loaded to form Ul/Ag/Cu-NCMs, the shape and size changed to a hexagonal shape, and they were well distributed in the solution with a size range of 10–35 nm (Figure 2G and Table 2). The image denotes polydispersed Ul/Ag/Cu-NCMs with a frequency in the range of 15 to 20 nm with 39%. The image demonstrates there is a core shell around the UL/Ag_2_ O-NPS, Ul/CuO-NPs, and Ul/Ag/Cu-NCMs; the shell appears faint and dark around the nanoparticles and is denoted by arrows in the images. The shell may be related to organic compounds derived from *U. lactuca.*

The core-shell nanomaterials can be extended to the class of nanomaterials having distinct boundary materials covering (either fully or partially) the inner component. Core-shell nanoparticles (core-shell NPs) are structures that frequently combine the characteristics of the two (or more) materials used, with the shell controlling the surface properties of the particles while encasing the core entirely [67].

The results indicate that *U. lactuca* is an excellent reducing and stabilizing agent. López-Ubaldo et al. [68] reported the size of Ag/Cu-NCMs biosynthesized by *Ricinus communis* was in the range of 10–25 nm and spheroid in shape. The size of Ag/Cu-NCMs green synthesized by *Opuntia ficusindica*, as indicated by Rocha-Rocha et al. [69], ranged from 10 nm to 20 nm. Mohamad et al. [70] stated that leaf palm extract efficiently reduced and stabilized agents for the biosynthesis of Cu/Ag-Nano composites. The supernatant of *Rhodopseudomonas capsulate* showed high efficiency when it was used to synthesize Cu/Ag-NCMs.

#### 2.1.3. Energy Dispersive X-ray (EDX) of UL/Ag_2_ O-NPS, Ul/CuO-NPs, and Ul/Ag/Cu-NCMs Derived from *U. lactuca*

**Figure 3:**
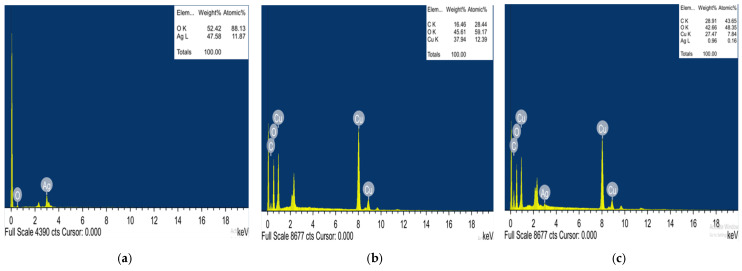
Energy dispersive X-ray (EDX) of UL/Ag_2_ O-NPS (**a**), Ul/CuO-NPs (**b**), and Ul/Ag/Cu-NCMs (**c**) derived from *U. lactuca*.

Energy dispersive X-ray (EDX) is a vital analytical procedure used to verify the sample’s elemental composition. Ag and O were found, by weight, in UL/Ag_2_ O-NPS at 54.42 and 47.58%, respectively. The chemical composition of the prepared Ul/CuO-NPs was identified by EDX analysis, as shown in Figure 3b. The EDX analysis of the Ul/CuO-NPs revealed that there was 37.94% copper, 45.61% oxygen, and 16.46% carbon. The four peaks are C, O, Cu, and Ag, with weight percentages of 28.91, 42.66, 27.47 and 0.96, respectively in Ul/Ag/Cu-NCMs. The extensive presence of Cu over Ag may be related to the methods of synthesis that use 1:9 Ag:Cu in the synthesis of nanocomposites. The appearance of different elements, such as oxygen 42.66% and carbon 17.08%, may be due to the organic compounds found in the alga extract [71].

#### 2.1.4. X-ray Diffraction (XRD) of Ul/Ag_2_ O-NPS, Ul/CuO-NPs, and Ul/Ag/Cu-NCMs Derived from *U. lactuca*

**Figure 4:**
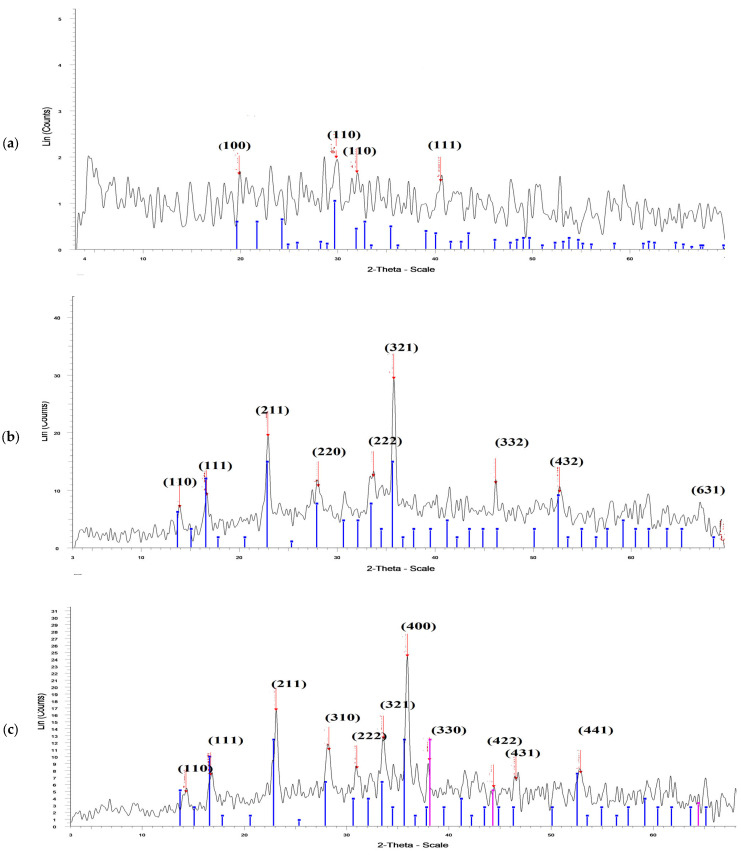
X-ray diffraction of UL/Ag_2_ O-NPS (**a**), Ul/CuO-NPs(**b**), and Ul/Ag/Cu-NCMs (**c**) derived from *U. lactuca*.

X-ray diffraction was used to investigate the size and crystallization of UL/Ag_2_ O-NPS synthesized by *U. lactuca*. The results revealed the peak positions with 2Ө values of 19.843, 29.841, 31.972, and 40.6. The Miller indices (h k l) for each peak were indexed as 100, 110, 110, and 111, respectively (Figure 4a, and Table 3a). These results confirm the formation of UL/Ag_2_ O-NPS as nanocrystals, and the crystal size ranged from 12.2 to 36.7. The major crystalline peak was obtained at 2 theta 29.841 with an intensity of 100% and a size of 12.2 nm. The X-ray diffraction of the Ag-NPs biofabricated by *Turbinaria urbinate* showed two intense peaks at 27.94 and 32.27, which correspond to Miller indices of 110 and 111 [72]. Five peaks of X-ray diffraction patterns were obtained with biofabricated Ag-NPs obtained by marine green alga *Ulva fasciata*, with 2Ө values of 27.925, 32.409, 46.333, 57.582, and 76.618 degrees corresponding to 111, 200, 220, 222, and 331, respectively [73]. The X-ray diffraction peaks of gelatin-capped Ag-NPs biofabricated by *Oscillatoria limnetica* extract had 2Ө values of 14.26, 24.15, 30.08, 32.02, and 42.01°, which correspond with Miller indices of 100, 100, 110, 110, and 111, respectively [74].

The XRD spectra of Ul/CuO-NMPs derived from *U. lactuca* reveal the crystal lattice structure of the biosynthesized Ul/CuO-NPs (Figure 4b and Table 3b). The XRD spectrum analysis for Ul/CuO-NPs shows diffraction peaks at 2Ө 13.865, 16.608, 22.889, 28.035, 33.673, 35.763, 46.167, 52.718, and 69.226; these diffraction peaks were indexed as 110, 111, 211, 220, 222, 321, 332, 432, and 631, respectively. The results demonstrate that the Ul/CuO-NPs is crystalline, and the crystal size ranged from 8.2 to 33. The major crystalline peak was obtained at 2 theta 35.763 with an intensity of 100%, and a size of 21.3 nm. The peak positions of Ul/CuO-NPS synthesized by macro green alga *Ulva fasciata* were at 2Ө values of 16.36, 22.62, 27.77, 31.74, 35.43, 41.14, 52.37, 56.07, 60.02, 66.55, and 73.64, which were indexed as 100, 110, 111, 200, 320, 210, 211, 221, 310, 311, 222, and 320, respectively [57]. The diffraction peaks of Cu-NPs synthesized by red alga *Pterocladia capillacea* were at 2Ө 10.9, 27.1,31.5, 45.2,56.3, and 75.1°, and were indexed (h k l) as 135, 114, 375, 220, and 105, respectively [75]. The XRD patterns of the Cu-NPs biofabricated by a soluble polysaccharide extracted from marine alga *Sargassum vulgare* were at 2Ө 33.8, 39.7, 47.3, 65.7, and 78.8° indexed with Miller plans as 110,111, 200, 220, and 313, respectively [76]. The results in Figure 4c and Table 3b show the peak positions of the XRD patterns of Ul/Ag/Cu-NCMs derived from *U. lactuca*, with 2Ө values of 14.22, 16.65, 23.05, 28.23, 30.98, 33.57, 35.91, 38.09, 44.38, 46.57, and 52.92; the Miller indices (h k l) for each peak were indexed as 110, 111, 211, 310, 222, 321, 400, 330, 422, 431, and 441, respectively. The most intense 100% peak was obtained at 2 theta 35.914 and size 28.8. The results denote the crystalline UL/Ag_2_ O-NPS, Ul/CuO-NPs, and Ul/Ag/Cu-NCMs derived from *U. lactuca*.

#### 2.1.5. Zeta Potential of UL/Ag_2_ O-NPS, Ul/CuO-NPs, and Ul/Ag/Cu-NCMs Derived from *U. lactuca*

**Figure 5:**
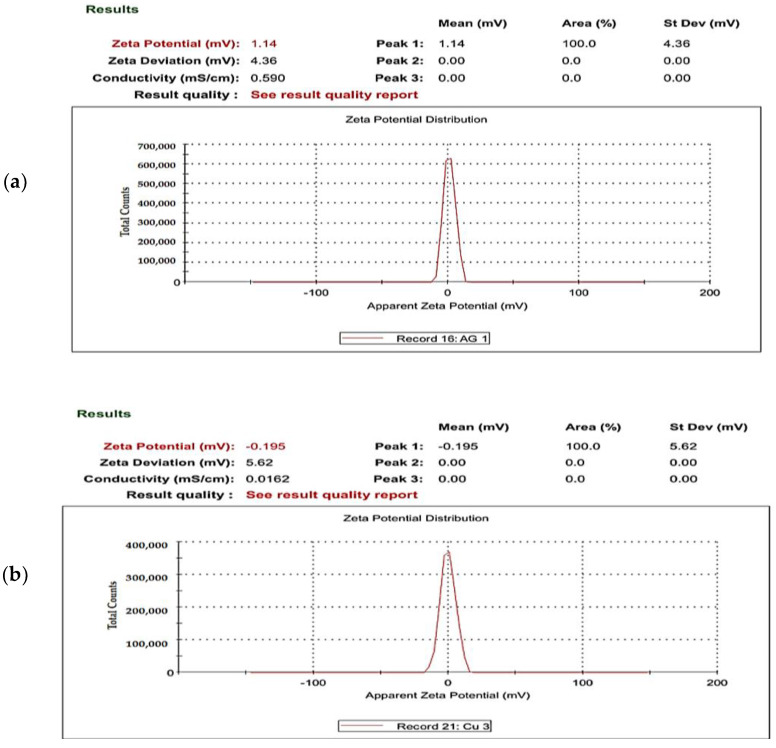

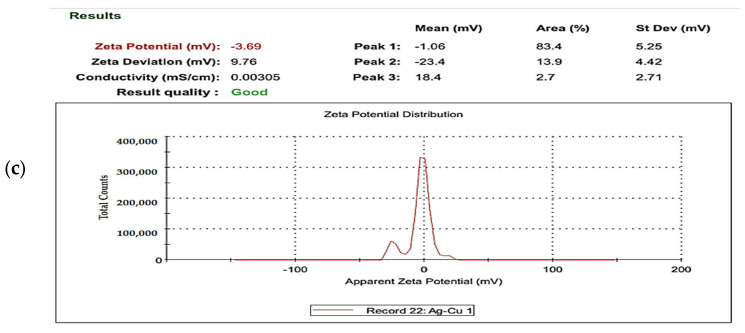
Zeta potential of UL/Ag_2_ O-NPS (**a**), Ul/CuO-NPs (**b**), and Ul/Ag/Cu-NCMs (**c**) derived from *U. lactuca*.

The zeta potential is an analysis method that measures the number of electric charges on the nanoparticle’s surface. Figure 5 demonstrates the zeta potential of UL/Ag_2_ O-NPS, Ul/CuO-NPs, and Ul/Ag/Cu-NCMs derived from *U. lactuca.* The results show that the zeta potential value of UL/Ag_2_ O-NPS has a positive charge (+1.14), while the zeta potential values of Ul/CuO-NPs and Ul/Ag/Cu-NCMs have negative charges (−0.195 and −3.69, respectively). The zeta potential values differ according to the methods used and plant synthesis. The silver nanoparticles formed by the fungus *Trichoderma viride* had a positive charge [77]. The zeta potential values of the Ag-NPs produced by photosynthesis by *Oscillatoria limnetica* had a highly positive charge [78].

### 2.2. Antibacterial Activity of Synthesized UL/Ag_2_ O-NPS, Ul/CuO-NPs, and Ul/Ag/Cu-NCMs Derived from U. lactuca

**Figure 6:**
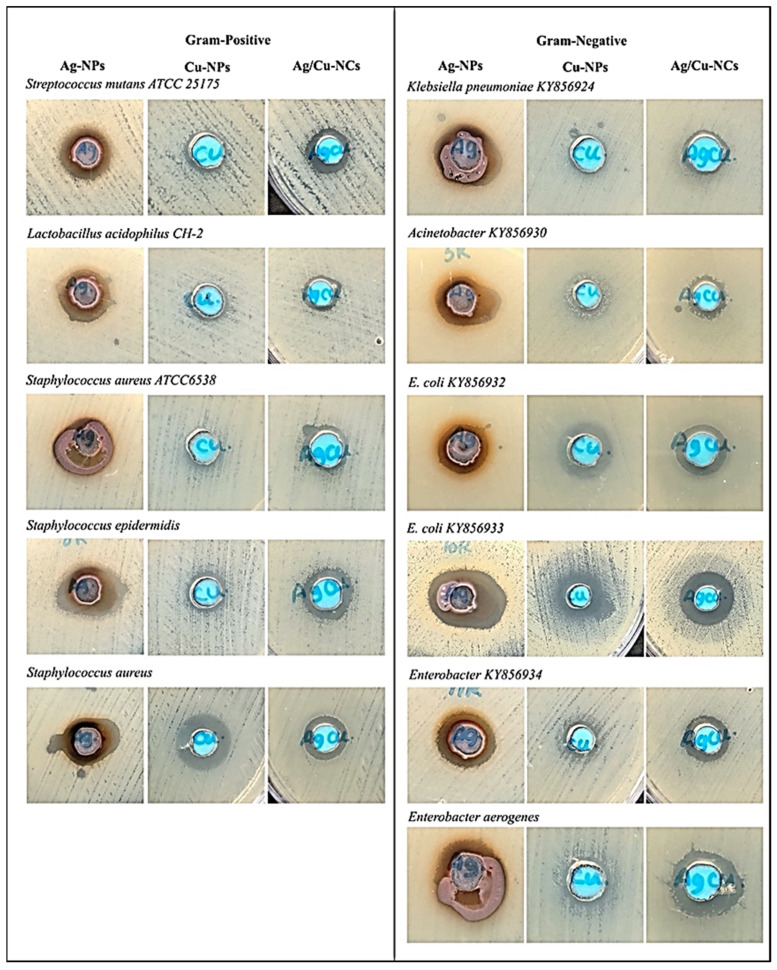
Antibacterial effects of green synthesized UL/Ag_2_ O-NPS, Ul/CuO-NPs, and Ul/Ag/Cu-NCMs (100 μL/well).

The antibacterial activity of UL/Ag_2_ O-NPS, Ul/CuO-NPs, and Ul/Ag/Cu-NCMs derived from *U. lactuca* in different volumes (10, 30, and 100 µL at the same concentration of 1 mg/mL) was tested against 11 bacterial strains of Gram-negative and Gram-positive multidrug-resistant bacteria: *Streptococcus mutans* ATCC 25175 and *Lactobacillus acidophilus* CH-2 [79]. The results indicate that the different volumes and types of nanoparticles had significant effects on the antibacterial activity. The higher volumes (100 µL/well) possessed higher antibacterial activities than 30 and 10 µL/well (Figure 6 and Table 4). The maximum zone of inhibition for the volume (100 μL) of Ul/Ag_2_ O-NPs was recorded against *Streptococcus mutans* ATCC 25175 (17 mm), followed by Ul/Ag/Cu-NCMs (14 mm). No significant effect of Ul/CuO-NPs (100 μL) and Ul/Ag-NPs with volume of 10 and 30 µL were noted. The lowest inhibition zone was recorded with Ul/CuO-NPs (10 and 30 µL). The UL/Ag_2_ O-NPS showed the highest antibacterial activity (18.3 mm at 100 µL) against *Lactobacillus acidophilus* CH-2, followed by Ul/Ag/Cu-NCMs (16 mm at 100 µL). The Ul/CuO-NPs showed the lowest antibacterial activity against *Staphylococcus aureus* ATCC6538 and *Staphylococcus aureus* in the inhibition zone (7 mm at 10, and 30 µL). The Ul/Ag-NPs and Ul/Cu-NPs possessed better antibacterial activity against *Staphylococcus aureus* than the Ul/Ag/Cu-NCMs with a volume of 100 µL. The Ul/Ag_2_ O-NPs denoted the highest antibacterial activity against *Klebsiella pneumoniae* KY856924, *Acinetobacter* KY856930, *E. coli* KY856933, and *Enterobacter* KY856934 at 100 µL/well, but the results showed that the Ul/CuO-NPs had the highest antibacterial activity against *E. coli* KY856932, and Ul/Ag/Cu-NCMs had higher activity against *Enterobacter aerogenes* at 100 µL/well. This study demonstrates that the UL/Ag_2_ O-NPS had more antibacterial activity in most tested bacteria, except *E. coli* KY856932 and *Enterobacter aerogenes*. The higher antibacterial activity of UL/Ag_2_ O-NPS over Ul/CuO-NPs and Ul/Ag/Cu-NCMs may be due to Ul/Ag_2_ O-NPs having a positive charge. The positively charged Ag-NPs displayed the largest inhibitory zones against all the strains tested and in all the concentrations employed. In any concentration examined, neutral and negatively charged AgNPs did not exhibit enough antibacterial action against *P. vulgaris* [80]. The positively charged BPEI-AgNPs were the most toxic NPs, whereas the more negatively charged citrate-AgNPs were the least hazardous [81]. Bacteria with a negative surface charge come into contact with nanoparticles with a positive zeta potential, and the electrostatic forces that result encourage a stronger attraction and interaction between the two organisms and may even allow for bacterial membrane penetration [82]. Also possible are the low-release Ul/CuO-NPs and Ul/Ag/Cu-NCMs. The Cu-NPs may possess antibacterial activity by denaturing the proteins and enzymes of bacteria [83]. An investigation was conducted by Chandraker et al. [84] and an excellent result was seen with a 12.43 mm zone of inhibition for the antibacterial activity of green Cu-NPs produced utilizing an aqueous leaf extract of *Ageratum houstonianum* against the Gram-negative bacterium *Escherichia coli*. A strong antibacterial effect of Cu-NPs on Gram-positive *Bacillus subtilis* and Gram-negative *Escherichia coli* was also mediated by an aqueous extract of *Curcuma longa*. When compared to Gram-positive bacteria, the Gram-negative bacteria had a greater zone of inhibitory growth, which suggests that the Gram-positive bacteria are more sensitive to Cu-NPs [85]. A previous study performed by Jayandran et al. [86] compared the antibacterial activities of Cu-NPs synthesized using curcumin against two Gram-positive (*Staphylococcus aureus* and *Bacillus subtilis*) and Gram-negative (*Escherichia coli*) bacteria to the antibacterial activity of pure curcumin. Fascinatingly, the zone of inhibition recognized for Cu-NPs against *S. aureus* and *B. subtilis* showed higher antibacterial activity than the normal drug, chloramphenicol.

### 2.3. Minimum Inhibitory Concentration (MIC) and Minimum Bactericidal Concentration (MBC) Assays

The MIC value can be used to assess the effectiveness of nanomaterials. The MIC is described as the lowest antimicrobial compound concentration that inhibits a microorganism’s growth [87]. The results in Table 5 demonstrate that, after 24 h of incubation at 37 °C, the MIC value of UL/Ag_2_ O-NPS was 0.062 mg/L with *Streptococcus mutans* ATCC 25175, *Lactobacillus acidophilus* CH-2, *E. coli* KY856933, and *Enterobacter* KY856934. The MIC value (0.25 mg/mL of UL/Ag_2_ O-NPS) was observed with *Lactobacillus acidophilus* CH-2, *Staphylococcus aureus* ATCC 25923, *Klebsiella pneumoniae* KY856924, *Acinetobacter* KY856930, *E. coli* KY856932, *E. coli* KY856933, *Enterobacter* KY856934, and *Enterobacter aerogenes*. The MIC values of Ul/Ag/Cu-NCMs were 0.25 mg/mL with *Streptococcus mutans* ATCC 25175, *Lactobacillus acidophilus* CH-2, *Staphylococcus aureus* ATCC6538, *Klebsiella pneumoniae* KY856924, *Acinetobacter* KY856930, *E. coli* KY856932, *Enterobacter* KY856934, and *Enterobacter aerogenes.* The MBC is the lowest antibacterial agent concentration that kills most bacterial inoculums [88]. The results in Table 5 demonstrate the MBC of UL/Ag_2_ O-NPS, Ul/CuO-NPs, and Ul/Ag/Cu-NCMs. The results show that the MBC of UL/Ag_2_ O-NPS was 0.125 mg/mL with *Streptococcus mutans* ATCC 25175, *Lactobacillus acidophilus* CH-2, *Staphylococcus epidermidis* ATCC 12228, *E. coli* KY856933, and *Enterobacter* KY856934. The MBC was 0.5 mg/mL of Ul/CuO-NPs and Ul/Ag/Cu-NCMs with *Streptococcus mutans* ATCC 25175, as well as of Ul/Ag/Cu-NCMs with *Staphylococcus aureus* ATCC 25923 and of UL/Ag_2_ O-NPS with *Klebsiella pneumonia* KY856924 and *E. coli* KY856932. The MBC values were more than 1 mg/mL of Ul/CuO-NPs with *Lactobacillus acidophilus* CH-2, *Staphylococcus aureus* ATCC 25923, *Staphylococcus epidermidis*, *Klebsiella pneumoniae* KY856924, *Acinetobacter* KY856930, *E. coli* KY856932, and *Enterobacter* KY856934; the MBC values were also more than 1 mg/mL of Ul/Ag/Cu-NCMs with *Staphylococcus aureus* ATCC6538, *Staphylococcus epidermidis* ATCC 12228, *Klebsiella pneumoniae* KY856924, *Acinetobacter* KY856930, *E. coli* KY856932, *Enterobacter* KY856934, and *Enterobacter aerogenes*. It was revealed that the Ag-NPs had a more vital antibacterial capacity than the Cu-NPs, suggesting that Ag ions are more effective antimicrobial agents than Cu ions [89,90]. The Ag-NPs also exhibited greater antibacterial potency against different strains of *E. coli* and *S. aureus*, as well as against fungi [91], which may be a result of their more potent interaction with the polysaccharides and proteins found on cell walls [92]. Because Cu-NPs have a lower antibacterial capacity than Ag-NPs, the possibility of an oxide layer on them has been suggested [93,94].

### 2.4. Biofilm Formation Inhibition Assay

**Figure 7:**
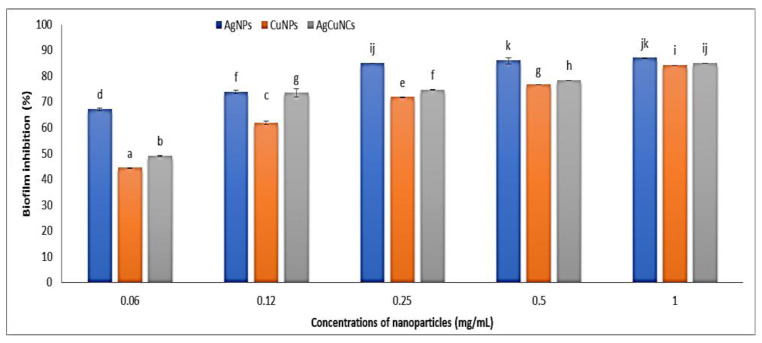
Anti-biofilm studies of green synthesized Ul/Ag_2_ ONPS, Ul/CuO-NPs, and Ul/Ag/Cu-NCMs in the presence of biofilm-forming of *E. coli* KY856933. Different letters denote the significant values of mean; bars are St-error.

A static biofilm assay was performed to evaluate the effect of the synthesized UL/Ag_2_ O-NPS, Ul/CuO-NPs, and Ul/Ag/Cu-NCMs derived from *U. lactuca* on *E. coli* KY856933 biofilm formation, and showed positive biofilm formation before treatment with the nanoparticles. Figure 7 shows the biofilm inhibition percentage at different concentrations of nanoparticles (1, 0.5, 0.25, 0.12, and 0.06 mg/mL). The results obtained in the microtiter plates show that the highest inhibition percentage was 87.02 with 0.5 mg/mL UL/Ag_2_ O-NPS, followed by 1.0 mg/mL UL/Ag_2_ O-NPS (86.31%). The same inhibition percentage was obtained with 0.25 mg/mL UL/Ag_2_ O-NPS, and with 1.0 mg/mL Ul/Ag/Cu-NCMs, the inhibition was 85.5%. The lowest percentage of biofilm inhibition was 44.38 with 0.06 mg/mL Ul/CuO-NPs. The order of anti-biofilm strength formed by *Escherichia coli* ATCC 25922, *Pseudomonas aeruginosa* ATCC 27853, and *Staphylococcus aureus* ATCC 43300 with biosynthesis nanocomposite was Ag–TiO_2_  >  TiO_2_–Ag  >  Cu–Ag  >  Ag–Cu [95]. The bio-Ag-NPs inhibited biofilm formation by both *Escherichia coli* and *S. aureus* at concentrations of 4 μg/mL [96]. The Ag-NPs demonstrated notable anti-biofilm activity against *S. aureus* biofilms and powerful bactericidal effects against *P. aeruginosa* and *S. aureus*, respectively [97]. The minimal inhibitory concentration of the produced Ag-NPs against biofilm-forming *Staphylococcus aureus* verified their concentration-dependent inhibition (MIC) [98].

## 3. Material and Methods

### 3.1. Extraction of Algae

**Figure 8:**
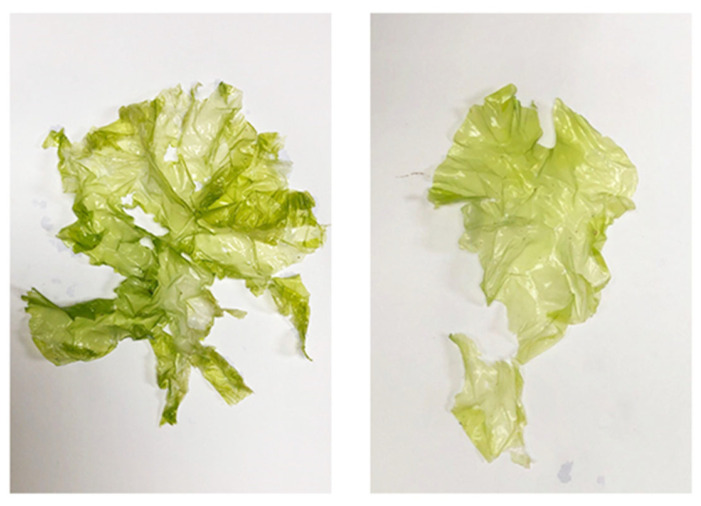
Morphological shape of *Ulva lactuca* (collected from seashore or Red Sea, Jeddah, Saudi Arabia).

At the end of January, in the winter, *Ulva lactuca* (Figure 8) was gathered from the seashore (21°.637086 N and 39°.101631 E) in Jeddah, Saudi Arabia (2020). One gram of air-dried *U. lactuca* was mixed with 100 mL of deionized (D.D.) water to create the Ag-NPS and Ag–Cu NCMS. This mixture was then boiled for an hour and filtered, and the remaining 100 mL of D.D. water was added. For the CuO-NPs, 100 mL of D.D. water was combined with six grams of air-dried *U. lactuca* alga, boiled for one hour, filtered, and then the remaining 100 mL of D.D. water was added.

### 3.2. Synthesis of Silver/Copper Nanocomposites (Ul/Ag/Cu-NCMs) by U. lactuca Extract

Briefly, 60 mL of distilled and deionized water were combined with 9 mM of copper II sulfate and 1 mM of silver nitrate (D.D. water). Drop by drop, 40 mL of aqueous algal extract was added to the Ag/Cu solution, followed by 0.2 M NaOH, which produced a grayish precipitate that was left in a magnetic stirrer for 2 h [70]. To remove the contaminants, the mixture was centrifuged, the supernatant was eliminated, and the grayish precipitate was washed twice with 50 mL of D.D. water and once more with 50 mL of ethanol by repeated centrifugation. After one day of air drying, silver/copper nanoparticle powder was produced.

### 3.3. Biosynthesis of Silver Nanoparticles Using Alga Aqueous Extract

Alga extract (30 mL) was injected drop by drop with a syringe on 70 mL of 1 mM AgNO_3_ on a magnetic stirrer until the color changed from clear to brown to darker brown, after ten minutes.

### 3.4. Biosynthesis Copper Oxide Nanoparticles (Ul/CuO-NPs) by U. lactuca Extract

A faint blue powder of copper nanoparticles was obtained after air drying for one day. Copper II sulfate, CuSO_4_ (9 mM), was added to 70 mL distal and deionized water (D.D. water) under constant stirring. Thirty ml of aqueous algal extract was added drop by drop to the copper solution. After 10 min of stirring, 0.2 M NaOH was added, changing the color from blue to greenish; this was then placed in a magnetic stirrer for 2 h [99]. The greenish precipitate was filtered and washed twice with 50 mL of D.D. water and then with 50 mL of ethanol to eliminate the impurities.

### 3.5. Structural and Morphological Characterization

The synthesized UL/Ag_2_ O-NPS, Ul/Ag/Cu-NCMs, and Ul/CuO-NPs were dried at 60 °C, and characterized by the following devices: scanning electron microscopy (SEM) (30 kV (SEM, JEOL JSM-6510/v, Tokyo, Japan); transmission electron microscopy (TEM) (JEOL JSM-6510/v, Tokyo, Japan); and X-ray diffraction (XRD) (PAN Analytical X-Pert PRO, spectris plc, Almelo, The Netherlands). According to the Scherrer equation, the mean particle diameters of the nanoparticles were calculated from the XRD pattern:

D = Kλ/βCOSθ

K is constant; λ is the wavelength of the X-ray (1.54060 Å); and β is the full width at half-maximum (FWHM).

Fourier transform infrared (FTIR) spectroscopy (Thermo Fisher Nicolet IS10, (Waltham, MA, USA)), zeta potential and energy dispersion (Malvern Zeta size Nano-Zs90, Malvern, PA, USA), and X-ray spectroscopy (EDX) (JEOL JSM-6510/v, Tokyo, Japan) were also performed.

### 3.6. Antibacterial Assay

The antibacterial activity of UL/Ag_2_ O-NPS, Ul/Ag/Cu-NCMs, and Ul/CuO-NPs was investigated against eleven pathogenic bacterial strains. Six Gram-negative bacteria (*Klebsiella pneumoniae* KY856924, *Acinetobacter* KY856930, *E. coli* KY856932, *E. coli* KY856933, *Enterobacter* KY856934, and *Enterobacter aerogenes*), which were isolated from different medical samples and identified as multidrug-resistant in a previous study [78, 100], in addition to five pathogenic Gram-positive control strains (*Klebsiella pneumoniae* KY856924, *Staphylococcus aureus* ATCC 6538, *Staphylococcus aureus* ATCC 25923, *Staphylococcus epidermidis* ATCC 12228, and *Streptococcus mutans* ATCC 25175) were utilized. All the bacterial strains were cultured in nutrient broth at 37 °C for 24 h. The bacterial strains were spread on Mueller–Hinton agar (MHA) using a sterile cotton swab. Wells were made in the agar plates and filled with 10, 30, and 100 µL of 20 mg/mL UL/Ag_2_ O-NPS, Ul/Ag/Cu-NCMs, and Ul/CuO-NPs, respectively. The plates were incubated at 37 °C for 24 h, and the zone of inhibition was observed after 24 h of incubation.

### 3.7. Minimum Inhibitory Concentration (MIC) Assays

The minimum inhibitory concentrations (MIC) of green synthesized nanoparticles were determined in a 24-well culture plate using standard broth micro-dilution methods to verify the antibacterial activity. Two-fold dilutions of UL/Ag_2_ O-NPS, Ul/Ag/Cu-NCMs, and Ul/CuO-NPs in concentrations ranging from 1 mg/mL to 0.062 mg/mL were prepared to determine the MIC in the nutrient broth media. The results were observed and compared to the negative control after 24 h at 37 °C of incubation.

### 3.8. Minimum Bactericidal Concentration (MBC) Assays

After the MIC determination of the nanoparticles, the MBC was conducted by transferring 10 μL from each overnight MIC culture plate well to sterile Mueller–Hinton agar (MHA) fresh plates. Viable growth was determined after 24 h at 37 °C. The lowest concentration with no visible growth on the MHA plate was recorded as the MBC value.

### 3.9. Biofilm Formation Inhibition Assay

The biofilm inhibition assay was performed as described previously by Heikens et al. [101] and Hamed et al. [102] with the following modifications: one colony of overnight plate-grown *E. coli* KY856933 bacterial culture (which showed positive biofilm formation on Congo red agar) was resuspended in 5 mL tryptic soy broth (TSB) supplemented with 0.25% glucose on a shaking incubator at 37 °C to an OD of 660.

The bacterial cultures were diluted with fresh media (1:20) and supplemented with different concentrations (1, 0.5, 0.25, 0.12, and 0.06 mg/mL) of UL/Ag_2_ O-NPS, Ul/CuO-NPs, and UL/Ag/Cu-NMCs separately, in addition to a culture without nanoparticles as a positive control. Then, 200 µL of each concentration was aliquoted into the wells of a 24-well flat-bottom polystyrene microtiter plate and incubated for 24 h at 37 °C without agitation. After incubation, the bacteria were removed, the wells were washed with 200 µL phosphate buffer saline (PBS), and the plates were dried for 1 h at room temperature.

A total of 100 mL of 0.2% Gram’s crystal violet was added to each well for 15 min after the first hour. After the stain was eliminated, 200 µL of PBS were used to wash the plates three times. At room temperature, the plates were dried for 15 min. Each well received one milliliter of ethanol (95%) before being incubated for 15 min. At 570 nm, the reaction mixture was spectrophotometrically measured.

$$\%\;\mathrm{of}\;\mathrm{inhibition}=\left(\frac{\mathrm{OD}\;\mathrm{in}\;\mathrm{control}\;-\;\mathrm{OD}\;\mathrm{in}\;\mathrm{treatment}\;}{\;\mathrm{OD}\;\mathrm{in}\;\mathrm{control}}\right)*100$$

### 3.10. Statistical Analysis

The experiments were performed in triplicate and the results were considered as the means ± standard error of the mean. An analysis of variance (ANOVA) was performed on all of the data. Duncan’s multiple range tests were used to determine the significance of the variable mean differences (*p* ≤ 0.05), SPSS version 16.

## 4. Conclusions

In this experimental study, *Ulva lactuca* aqueous extract was successfully used to synthesize silver and copper nanoparticles and their composites. Their characterization was achieved using FTIR, SEM, TEM, EDX, XRD, and the zeta potential. The FTIR analysis reveals that the functional groups of nanoparticles and their composites differ. The TEM image demonstrates changes in the shape and size of the silver and copper nanoparticles and their composites, which were biofabricated by green alga *U. lactuca.* The zeta potential denotes that the UL/Ag_2_ O-NPS have a positive charge, while the Ul/CuO-NPs and Ul/Ag/Cu-NPs have a negative charge. The fact that the UL/Ag_2_ O-NPS were more effective against the tested pathogenic bacteria than both the Ul/CuO-NPs and Ul/Ag/Cu-NPs may be due to the positive charge of UL/Ag_2_ O-NPS, as well as the small size of the nanoparticles. The inhibition of biofilm formation by *E. coli* KY856933 was the highest with the applied UL/Ag_2_ O-NPS, followed by the Ul/CuO-NPs and Ul/Ag/Cu-NMPs.

## Figures and Tables

**Table 1:** Absorption peaks assigned to the active groups of Ul/Ag_2_O-NPS, Ul/Ag/Cu-NCMs, and Ul/CuO-NPs derived from *U. lactuca*.

Wavenumber cm^−1^	Ul/Ag/Cu-NCMs	Ul/CuO-NPs	UL/Ag_2_ O-NPS	Assignment	References
3752	Nd	Nd	D	OH bonds	[38]
3588	D	−1	Nd	OH bonds	[39]
3565	D	−1	Nd	OH bonds	[40]
3419	Nd	Nd	D	Bonded N–H/C–H/O–H stretching of amines and amides	[41]
3390	D	D	Nd	O–H Stretching	[42]
2925	D	+2	Nd	CH stretching bands	[43]
2426	Nd	Nd	D	Carboxyl acid	[41]
1765	Nd	Nd	D	Carbonyl stretching C=O	[44]
1633	D	+2	+3	N–H stretching band	[45]
1386	Nd	D	−3	C–H stretching	[46]
1202	Nd	Nd	D	Alkyl amine	[47]
1125	D	−2	Nd	Amide III band region	[48]
1088	D	+2	−9	C–N stretching absorption of primary aliphatic amines	[49]
1025	D	Nd	Nd	Aromatic C–H in plane deformation	[50]
987	D	+1	Nd	Si–O stretching region	[51]
944	D	D	Nd	Phosphodiester region	[51]
874	D	+2	Nd	Organosulfate C–O–S	[52]
826	Nd	Nd	D	Stretching, C–C	[41]
780	D	+1	Nd	Pyridine (pyridine ring vibration and C–H deformation)	[53]
733	D	+1	Nd	Cu–O	[54]
631	D	+1	Nd	OH Stretching	[55]
600	D	+1	+6	Ring deformation of phenyl	[54]
509	D	Nd	Nd	Cu–O	[56]
483	D	D	Nd	Cu–O	[56]
417	D	+3	Nd	Cu–O	[56]

D: Detected; Nd: Not Detected; (−): shifted wavenumber by minus; (+): shifted wavenumber by addition.

**Table 2:** The main differences between biofabricated nanoparticles by *U. lactuca*.

Biofabricated Nanoparticles	Shape	Size Range (nm)	Size Range (nm) (Predominant)	Frequency%
UL/Ag_2_ O-NPS	Spherical	10–45	15–20	28%
Ul/CuO-NPs	Rod	10–40	20–25	50%
Ul/Ag/Cu-NCMs	Hexagonal	10–35	15–20	39%

**Table 3:** (a) X-ray diffraction of Ul/Ag_2_ O-NPS derived from *U. lactuca*. (b) X-ray diffraction of Ul/CuO-NPs derived from *U. lactuca*. (c). X-ray diffraction of Ul/Ag/Cu-NCMs derived from *U. lactuca*.

**(a)**					
**Peak position 2Ө**	**1000** × **sin^2^Ө**	**1000** × **sin^2^Ө/36**	**Reflection**	**Crystal size (nm)**	**Intensity %**
19.843	30	1	100	36.7	81.8
29.841	66	2	110	12.2	100
31.972	76	2	110	21.8	83.8
40.6	120	3	111	28.3	74.3
(**b**)					
13.865	15	2	110	24.1	23.7
16.608	22	3	111	20.9	30.8
22.889	40	6	211	28.4	66
28.035	59	8	220	0.7	36.1
33.673	85	12	222	8.2	42.1
35.763	95	14	321	21.3	100
46.167	153	22	332	14.7	38
52.718	199	29	432	10.5	32.7
69.226	327	46	631	33	0.9
(**c**)					
14.221	15	2	110	20.2	19.6
16.659	21	3	111	20	29.8
23.054	40	6	211	24.1	68.1
28.238	59	10	310	24.2	44.7
30.981	71	12	222	33.5	33.9
33.571	83	17	321	18.3	51.5
35.914	95	16	400	28.8	100
38.091	106	18	330	117.4	38.6
44.389	143	24	422	16.1	22.7
46.573	156	26	431	38.1	27.4
52.921	198	33	441	0.3	31.2

**Table 4:** Effect of different volumes (10, 30, 100 µL) of UL/Ag_2_ O-NPS, Ul/CuO-NPs, and Ul/Ag/Cu-NCMs (conc., 1 mg/mL) derived by *U. lactuca* in Gram-positive and Gram-negative bacteria, determined by inhibition zone (mm).

Bacterial Strains	Volume	UL/Ag_2_ O-NPS	Ul/CuO-NPs	Ul/Ag/Cu-NCMs	Significant
*Streptococcus mutans* ATCC 25175	10	11 ^a^ ± 0.28	7 ^a^ ± 0.28	9 ^abc^ ± 0.57	0.001
	30	11 ^a^ ± 1.15	7 ^a^ ± 1.15	9.5 ^bc^ ± 0.0	
	100	17 ^ded^ ± 1.15	11 ^a^ ± 0.28	14.5 ^hijk^ ± 1.15	
*Lactobacillus acidophilus* CH-2	10	9 ^a^ ± 0.5	7 ^a^ ± 0.57	7 ^a^ ± 0.28	0.001
	30	14 ^db^ ± 0.57	7 ^a^ ± 0..0	9.5 ^cd^ ± 0.28	
	100	18.3 ^def^ ± 0.57	11 ^b^ ± 0.57	16 ^k^ ± 0.57	
*Staphylococcus aureus* ATCC6538	10	10 ^a^ ± 0.0	7 ^a^ ± 0.0	7 ^a^ ± 0.57	0.001
	30	10 ^a^ ± 0.28	7 ^a^ ± 0.0	8 ^ab^ ± 0.57	
	100	17.5 ^def^ ± 1.15	11 ^b^ ± 0.57	13.5 ^ghi^ ± 0	
*Staphylococcus aureus*	10	10 ^a^ ± 0.57	7 ^a^ ± 0.0	9.5 ^bc^ ± 0.76	0.001
	30	10 ^a^ ± 0.0	7 ^a^ ± 0.0	9.5 ^bc^ ± 1.15	
	100	18.5 ^ef^ ± 0.0	17 ^d^ ± 0.57	13 ^fgh^ ± 1.15	
*Staphylococcus epidermidis*	10	9 ^a^ ± 0.28	7 ^a^ ± 0.0	9 ^abc^ ± 0.0	0.001
	30	11 ^a^ ± 0.28	8 ^a^ ± 0.57	10 ^bcd^ ± 0.57	
	100	19.3 ^f^ ± 0.57	12 ^b^ ± 0.57	14 ^hij^ ± 0.57	
*Klebsiella pneumoniae* KY856924	10	10 ^a^ ± 1.04	7 ^a^ ± 0.0	8 ^ab^ ± 0.86	0.001
	30	11 ^a^ ± 0.57	7 ^a^ ± 0.0	9 ^abc^ ± 0.0	
	100	16.5 ^cd^ ± 0.0	11 ^b^ ± 1.15	12 ^efg^ ± 0.57	
*Acinetobacter* KY856930	10	10 ^a^ ± 0.28	7 ^a^ ± 0.0	8 ^ab^ ± 0.57	0.001
	30	11 ^a^ ± 0.0	7 ^a^ ± 0.57	9 ^abc^ ± 0.28	
	100	17 ^de^ ± 1.15	10.^b^ ± 1.44	13 ^fgh^ ± 1.15	
*E. coli* KY856932	10	10 ^a^ ± 0	7 ^a^ ± 0.0	8 ^ab^ ± 0.0	0.001
	30	10 ^a^ ± 1.15	7 ^a^ ± 0.0	9 ^abc^ ± 1.15	
	100	14 ^b^ ± 0.57	15 ^c^ ± 0.750.0	14 ^hij^ ± 0	
*E. coli* KY856933	10	11 ^a^ ± 0	7 ^a^ ± 1.15	11 ^cde^ ± 0	0.001
	30	11 ^a^ ± 1.05	17 ^d^ ± 1.44	18 ^l^± 0.57	
	100	21.3 ^g^ ± 0.57	20.5 ^e^ ± 0.0	18 ^l^ ± 0.0	
*Enterobacter* KY856934	10	9 ^a^ ± 0.57	7 ^a^ ± 0.0	9 ^abc^ ± 0.28	0.001
	30	17.^de^ ± 0.28	7 ^a^ ± 0.0	11.5 ^def^ ± 0.0	
	100	16 ^ab^ ± 0.0	15 ^c^ ± 0.0	15 ^c^ ± 01.15	
*Enterobacter aerogenes*	10	9 ^a^ ± 0.28	7 ^a^ ± 0.57	9 ^abc^ ± 0.28	0.001
	30	10 ^a^ ± 0.57	7 ^a^ ± 1.15	9 ^abc^ ± 0	
	100	15 ^ab^ ± 0.57	14 ^c^ ± 0.0	15.5 ^b^ ± 0.57	
Sig		0.001	0.001	0.001	

Different letters in each column are significant value (*p* ≤ 0.05).

**Table 5:** Minimum inhibitory concentration (MIC) assays and minimum bactericidal concentration (MBC) assays of UL/Ag_2_ O-NPS, Ul/CuO-NPs, and Ul/Ag/Cu-NCMs derived from *U. lactuca*.

Bacteria	Nano Types	MIC (Nano mg/mL)						MBC (Nano mg/mL)				
		1	0.5	0.25	0.125	0.062	0.03	1	0.5	0.25	0.125	0.062
*Streptococcus mutans* ATCC 25175	UL/Ag_2_ O-NPS	−	−	−	−	−	+	−	−	−	−	+
	Ul/CuO-NPs	−	−	−	+	+	+	−	−	+	+	+
	Ul/Ag/Cu-NCMs	−	−	−	+	+	+	−	−	+	+	+
*Lactobacillus acidophilus* CH-2	UL/Ag_2_ O-NPS	−	−	−	−	−	+	−	−	−	−	+
	Ul/CuO-NPs	−	−	−	+	+	+	+	+	+	+	+
	Ul/Ag/Cu-NCMs	−	−	−	+	+	+	−	−	−	+	+
*Staphylococcus aureus* ATCC6538	UL/Ag_2_ O-NPS	−	−	+	+	+	+	−	−	−	+	+
	Ul/CuO-NPs	−	−	−	+	+	+	+	+	+	+	+
	Ul/Ag/Cu-NCMs	−	−	−	+	+	+	+	+	+	+	+
*Staphylococcus aureus* ATCC 25923	UL/Ag2 O-NPS	−	−	−	+	+	+	−	−	−	+	+
	Ul/CuO-NPs	−	−	−	+	+	+	+	+	+	+	+
	Ul/Ag/Cu-NCMs	−	−	−	−	+	+	−	−	+	+	+
*Staphylococcus epidermidis* ATCC 12228	UL/Ag_2_ O-NPS	−	−	−	−	+	+	−	−	−	−	+
	Ul/CuO-NPs	−	−	−	+	+	+	+	+	+	+	+
	Ul/Ag/Cu-NCMs	−	−	−	−	+	+	+	+	+	+	+
*Klebsiella pneumoniae* KY856924	UL/Ag_2_ O-NPS	−	−	+	+	+	+	−	−	+	+	+
	Ul/CuO-NPs	−	−	−	+	+	+	+	+	+	+	+
	Ul/Ag/Cu-NCMs	−	−	−	+	+	+	+	+	+	+	+
*Acinetobacter KY856930*	UL/Ag_2_ O-NPS	−	+	+	+	+	+	−	+	+	+	+
	Ul/CuO-NPs	−	−	−	+	+	+	+	+	+	+	+
	Ul/Ag/Cu-NCMs	−	−	−	+	+	+	+	+	+	+	+
*E. coli* KY856932	UL/Ag_2_ O-NPS	−	−	+	+	+	+	−	−	+	+	+
	Ul/CuO-NPs	−	−	−	+	+	+	+	+	+	+	+
	Ul/Ag/Cu-NCMs	−	−	−	+	+	+	+	+	+	+	+
*E. coli* KY856933	UL/Ag_2_ O-NPS	−	−	−	−	−	+	−	−	−	−	+
	Ul/CuO-NPs	−	−	−	+	+	+	−	−	−	+	+
	Ul/Ag/Cu-NCMs	−	−	−	−	+	+	−	−	−	+	+
*Enterobacter KY856934*	UL/Ag_2_ O-NPS	−	−	−	−	−	+	−	−	−	−	+
	Ul/CuO-NPs	−	−	−	+	+	+	+	+	+	+	+
	Ul/Ag/Cu-NCMs	−	−	−	+	+	+	+	+	+	+	+
*Enterobacter aerogenes*	UL/Ag_2_ O-NPS	−	−	+	+	+	+	−	+	+	+	+
	Ul/CuO-NPs	−	−	−	+	+	+	+	+	+	+	+
	Ul/Ag/Cu-NCMs	−	−	−	+	+	+	+	+	+	+	+

Positive (+): turbidity, indicating growth; negative (−): no turbidity, indicating absence of growth.

## Data Availability

Not applicable.

## Sample Availability

Samples are available upon request from R.A.H.
